# The Identification of Intrinsic Chloramphenicol and Tetracycline Resistance Genes in Members of the *Bacillus cereus* Group (*sensu lato*)

**DOI:** 10.3389/fmicb.2016.02122

**Published:** 2017-01-04

**Authors:** Helen Glenwright, Susanne Pohl, Ferran Navarro, Elisenda Miro, Guillermo Jiménez, Anicet R. Blanch, Colin R. Harwood

**Affiliations:** ^1^Centre for Bacterial Cell Biology, Institute for Cell and Molecular Biology, Newcastle UniversityNewcastle upon Tyne, UK; ^2^Servei de Microbiologia, Hospital de la Santa Creu i Sant Pau, Institut d'Investigació Biomèdica Sant PauBarcelona, Spain; ^3^Research and Development Department, Rubinum S.A.Rubí, Spain; ^4^Department of Microbiology, University of BarcelonaBarcelona, Spain

**Keywords:** *Bacillus toyonensis*, gene knockouts, intrinsic antibiotic resistance, *Bacillus cereus*, quantitative PCR

## Abstract

*Bacillus toyonensis* strain BCT-7112^T^ (NCIMB 14858^T^) has been widely used as an additive in animal nutrition for more than 30 years without reports of adverse toxigenic effects. However, this strain is resistant to chloramphenicol and tetracycline and it is generally considered inadvisable to introduce into the food chain resistance determinants capable of being transferred to other bacterial strains, thereby adding to the pool of such determinants in the gastro-enteric systems of livestock species. We therefore characterized the resistance phenotypes of this strain and its close relatives to determine whether they were of recent origin, and therefore likely to be transmissible. To this end we identified the genes responsible for chloramphenicol (*catQ*) and tetracycline (*tetM*) resistance and confirmed the presence of homologs in other members of the *B. toyonensis* taxonomic unit. Unexpectedly, closely related strains encoding these genes did not exhibit chloramphenicol and tetracycline resistance phenotypes. To understand the differences in the behaviors, we cloned and expressed the genes, together with their upstream regulatory regions, into *Bacillus subtilis*. The data showed that the genes encoded functional proteins, but were expressed inefficiently from their native promoters. *B. toyonensis* is a taxonomic unit member of the *Bacillus cereus* group (*sensu lato*). We therefore extended the analysis to determine the extent to which homologous chloramphenicol and tetracycline resistance genes were present in other species within this group. This analysis revealed that homologous genes were present in nearly all representative species within the *B. cereus* group (*sensu lato*). The absence of known transposition elements and the observations that they are found at the same genomic locations, indicates that these chloramphenicol and tetracycline resistance genes are of ancient origin and intrinsic to this taxonomic group, rather than recent acquisitions. In this context we discuss definitions of what are and are not intrinsic genes, an issue that is of fundamental importance to both Regulatory Authorities, and the animal feed and related industries.

## Introduction

The *Bacillus cereus* group (*sensu lato*) forms an independent branch within the *Bacillus* genus and currently comprises eight closely related and validated species: *B*. *cereus* (*sensu stricto*), *B. anthracis, B. thuringiensis, B. mycoides, B. pseudomycoides, B. weihenstephanensis, B. cytotoxicus*, and *B. toyonensis* (Skerman et al., 1980; Lechner et al., 1998; Guinebretière et al., 2013; Jiménez et al., 2013). Three additional new species, *B. gaemokensis, B. manliponensis*, and *B. bingmayongensis*, have been recently reported to belong to *B. cereus* group, but their names are not currently validly published (Jung et al., 2010, 2011; Liu et al., 2014, 2015).

Until relatively recently, understanding the identification and phylogenetic relationships within the *B. cereus* group (*sensu lato*) has proved challenging because: (a) traditionally, taxonomic distinctions among this group have relied heavily on a small number of phenotypic traits that include, in some cases, genes encoded on plasmids (e.g., anthrax toxin, insecticidal toxins, immune-evading capsules) and morphological features (*B. mycoides*; Rasko et al., 2004); (b) 16S rRNA gene sequence data is not sufficient to differentiate bacteria within the group due to its high degree of conservation (Bavykin et al., 2004).

In a taxonomic study to determine whether *B. toyonensis* strain BCT-7112^T^, previously known as *B. cereus* var. *toyoi*, strain BCT-7112, was a new species within the *B. cereus* group, a polyphasic approach was performed in which both phenotypic and genotypic traits were analyzed (Jiménez et al., 2013). Taken together, the results indicated that strain BCT-7112^T^ did indeed represent a new species for which the name *B. toyonensis* sp. nov. was proposed and approved, with BCT-7112^T^ (=CECT 876^T^; = NCIMB 14858^T^) as the type strain (Oren and Garrity, 2014). The characteristics that distinguished strain BCT-7112^T^ from other *B. cereus* species were also shared by 10 other strains (Jiménez et al., 2013).

In a more recent taxonomic study, the affiliations of strains in the *B. cereus* group were established using a Genome BLAST Distance Phylogeny (GBDP) approach (Liu et al., 2015). This separated the 224 analyzed strains into 30 clusters; eleven known species-level clusters and 19 potentially novel species. In this analysis the 11 strains previously identified as belonging to the *B. toyonensis* taxonomic unit (Jiménez et al., 2013) formed a distinct cluster (BCG09) but was expanded to nineteen strains by the inclusion of eight additional strains. In the same study, an identical cluster was identified using a rapid typing method based on the *pycA* gene and, in separate studies, by multi-locus sequence analysis (MLSA) and whole-genome single nucleotide polymorphism (SNP)-based phylogeny (Böhm et al., 2015).

*B. toyonensis* BCT-7112^T^ has been widely used for more than 30 years as the active ingredient of Toyocerin®, an additive used in animal nutrition and its non-toxigenic nature has been shown in various studies (Williams et al., 2009; Trapecar et al., 2011; Blanch et al., 2014). However, it is resistant to chloramphenicol and tetracycline, a trait that is generally considered inadvisable for introduction into the food chain if associated with mobile genetic elements (MGEs). We therefore set out to identify the genes responsible for these resistance phenotypes and to establish whether they were intrinsic or recent acquisitions associated with horizontal gene transfer. Unexpectedly, we not only observed homologous genes in closely related strains, but in virtually all members of the *B. cereus* group (*sensu lato*), irrespective of their reported susceptibility to these antibiotics. We discuss our findings in the context of regulations governing the animal feed and related industries.

## Methods

### Strains and culture conditions

The strains and plasmids used in this study, together with their sources, are shown in Table 1. When required, the following antibiotic concentrations were used: for *E. coli*, 100 μg/ml ampicillin and 12.5 μg/ml chloramphenicol; for *Bacillus* strains, 1 μg/ml erythromycin, 100 μg/ml spectinomycin, and for chloramphenicol and tetracycline concentrations ranged from 1 to 32 μg/ml, as indicated in the text.

**Table 1:** **Strains and plasmids used in this study**.

**Strains**	Trait or relevant genotype^*^	Source/Reference^*^
***Bacillus cereus***		
ATCC 14579^T^		ATCC
***Bacillus thuringiensis***		
ATCC 10792		ATCC
***Bacillus toyonensis***		
BCT-7112^T^	*B. toyonensis* type strain, Tc^R^, Cm^R^	Rubinum S.A, Spain
BCT-7112Δtet	BCT-7112^T^ with the *tetM* gene replaced with a spectinomycin resistance gene, Cm^R^, Sp^R^, Tc^S^	This study
BCT-7112Δcat	BCT-7112^T^ with the *tetM* gene replaced with a spectinomycin resistance gene, Tc^R^, Sp^R^, Cm^S^	This study
Rock1-3	*B. toyonensis* strain isolated from soil in Rockville, Maryland. Previously *B. cereus* Rock1-3	US Naval Medical Research Center Zwick et al., 2012
Rock3-28	*B. toyonensis* strain isolated from soil in Rockville, Maryland. Previously *B. cereus* Rock3-28	US Naval Medical Research Center; Zwick et al., 2012
BCT-7112(pIGR1)	BCT-7112^T^ with pIGR, Tc^R^, Cm^R^, Em^R^	This study
BCT-7112Δtet(pIGR1)	BCT-7112Δtet with pIGR, Cm^R^, Sp^R^, Em^R^, Tc^S^	This study
BCT-7112Δcat(pIGR1)	BCT-7112Δcat with pIGR, Tc^R^, Sp^R^, Em^R^, Cm^S^	This study
Rock1-3(pIGR)	Rock1-3 with pIGR, Em^R^	This study
***Bacillus subtilis***		
168	*trpC2*	Institute Pasteur, Paris; Anagnostopoulos and Spizizen, 1961
168tetM7112	168 with *tetM* from BCT-7112^T^, Tc^R^	This study
168tetM1-3	168 with *tetM* from Rock1-3, Tc^R^	This study
168tetM3-28	168 with *tetM* from Rock3-28, Tc^R^	This study
168catQ7112	168 with *catQ* from BCT-7112^T^, Cm^R^	This study
168catQ1-3	168 with *catQ* from Rock1-3, Cm^R^	This study
168catQ3-28	168 with *catQ* from Rock3-28, Cm^R^	This study
***Escherichia coli***		
DH5α	*huA2 lac(del)U169 phoA glnV44 Φ80′ lacZ(del)M15 gyrA96 recA1 relA1 endA1 thi-1 hsdR*	Thermo Fisher Scientific
GM48	*dam^−^, dcm^−^*	Stratagene
**Plasmids**		
pUTE583	Cm^*R*^ in *E. coli*; Em^R^ and Ts in *B. toyonensis*	Theresa M. Koehler, The University of Texas: Chen et al., 2004
pUTEΔtetM	pUTE583 with *tetM* flanking regions and Sp^R^ cassette	This study
pUTEΔcatQ	pUTE583 with *catQ* flanking regions and Sp^R^ cassette	This study
pING1	pUTE583 with the *gerIC–nucB* intergenic region of plasmid pBCT77	This study
pDR111	*B. subtilis* integration/expression vector, Ap^R^ (*E. coli*), Sp^R^ (*B. subtilis*), *amyE*Δ, *lacI*, P_hyper-spank_	R. Daniel, Newcastle University
pDRtetM7112	pDR111 with *tetM* from BCT-7112^T^, Ap^R^, Sp^R^, Tc^R^	This study
pDRtetM1-3	pDR111 with *tetM* from Rock1-3, Ap^R^, Sp^R^, Tc^R^	This study
pDRtetM3-28	pDR111 with *tetM* from Rock3-28, Ap^R^, Sp^R^, Tc^R^	This study
pDRcatQ7112	pDR111 with *catQ* from BCT-7112^T^, Ap^R^, Sp^R^, Tc^R^	This study
pDRcatQ1-3	pDR111 with *catQ* from Rock1-3, Ap^R^, Sp^R^, Tc^R^	This study
pDRcatQ3-28	pDR111 with *catQ* from Rock3-28, Ap^R^, Sp^R^, Tc^R^	This study

* *ATCC, American Type Culture Collection; Ap^R^, ampicillin resistance; Cm^R^, chloramphenicol resistance; Em^R^, erythromycin resistance; Tc^R^, tetracycline resistance; Sp^R^, spectinomycin resistance*.

Minimum inhibitory concentration (MIC) experiments were carried out in accordance with the European Food Safety Authority's (EFSA) “Guidance on the assessment of bacterial antimicrobial susceptibility” (EFSA-FEEDAP, 2012) using the microdilution method. Solutions of the required antibiotics (0, 1, 2, 4, 8, 16, and 32 μg/ml) were made up in Brain Heart Infusion broth and 200 μl added to wells in a 96-well microtitre plate (MTP). The wells were inoculated with 20 μl of the test cultures, prepared from fresh colonies grown on Brain Heart Infusion agar. The MTP was incubated in a FLUOstar Optima plate reader for 18 h at 37°C with vigorous shaking, monitoring Optical Density (OD) at 600 nm. The experiments were carried out a minimum of three times and the representative data show on a linear rather than logarithmic graph to make it easier to compare growth profiles. The MIC was determined as the lowest concentration of the antibiotic for which no growth was detected.

### Construction of *tetM* and *catQ* knockout strains

**Figure 1:**
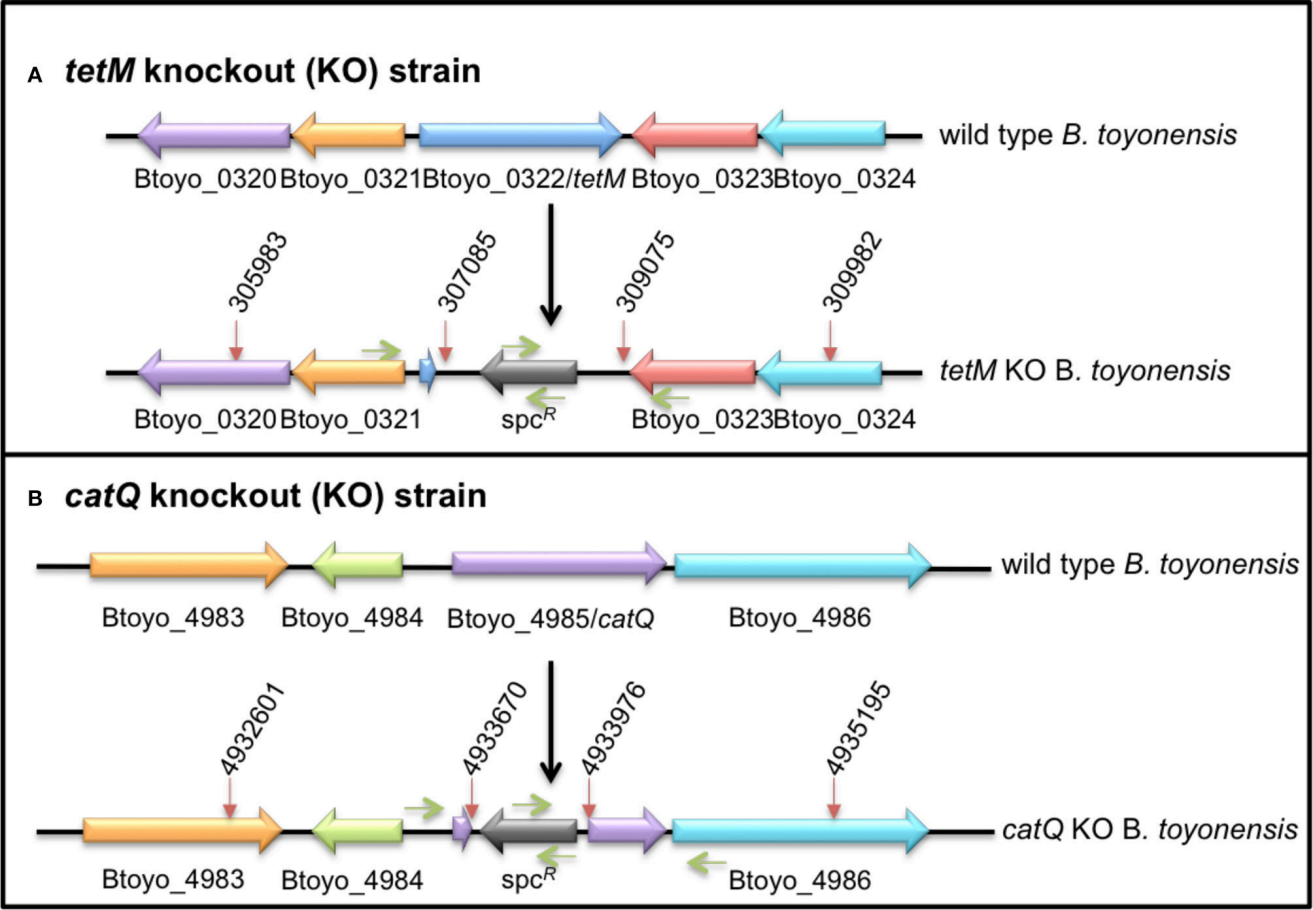
**Regions of the ***B. toyonensis*** BCT-7112^**T**^ genome containing (A)** tetracycline (Btoyo_0322, *tetM*) and **(B)** chloramphenicol (Btoyo_4985, *catQ*) resistance genes. The lower images show the locations (red arrows) of the upstream and downstream fragments and the spectinomycin resistance (*spc*^*R*^) gene that were used to replace the Btoyo_0322 or Btoyo_4985 genes by reciprocal recombination events, generating the *tetM* or *catQ* knockout strains BCT-7112Δtet and BCT-7112Δcat. The green arrows show the approximate locations of the primers used to sequence over the knockout regions.

In order to identify the genes responsible for tetracycline and chloramphenicol resistance in the genome of *B. toyonensis* BCT-7112^T^, a gene replacement strategy was employed. This involved the replacement of the target gene with a spectinomycin gene by a reciprocal recombination event. Briefly, two DNA knockout cassettes of ~3 kb were synthesized (DNA 2.0, Newark, California) and cloned into the vector pUTE583 (Koehler et al., 1994) to generate pUTEΔtetM and pUTEΔcatQ. Each synthesized fragment consisted of three components, a ~1000-bp region upstream of the target gene, a 947-bp spectinomycin resistance fragment and a ~1000-bp regions downstream of the target cassette. In the case of the *tetM* knockout cassette, a 1102-bp upstream fragment of the *B. toyonensis* BCT-7112^T^ chromosome (nucleotides 305983–307085) was fused to the 947-bp spectinomycin resistance fragment, which in turn was fused to the 907-bp downstream fragment of the *B. toyonensis* BCT-7112^T^ chromosome (nucleotides 309075–309983; Figure 1A). The *catQ* knockout cassette was similarly constructed with a 1069-bp upstream fragment (nucleotides 4932601–4933670) and 1219 downstream fragment (nucleotides 4933976–4935195) of the *B. toyonensis* BCT-7112^T^ chromosome, separated by the 947-bp spectinomycin resistance fragment (Figure 1B).

The bifunctional vector, pUTE853, is able to replicate in *E. coli* and members of the *B. cereus* group, but is lost from the latter at 37°C in the absence of antibiotic selection. The knockout plasmids, pUTEΔtetM and pUTEΔcatQ, were first passaged through a *dam-*minus *E. coli* strain (GM48) and then electro-transformed into *B. toyonensis* BCT-7112^T^, selecting for erythromycin resistance at 30°C (Koehler et al., 1994). The transformants were grown in the presence of erythromycin for 2 days, diluting 1:500 at the start and end of the day with fresh medium, and then shifted to 37°C for 2 days in the presence of spectinomycin, again diluting 1:500 at the start and end of the day with fresh medium. Samples were plated onto BHI agar containing spectinomycin, incubated overnight at 37°C, and individual colonies checked for erythromycin sensitivity and spectinomycin resistance, confirming the loss of the plasmid and integration of the knockout cassette into the chromosome. In each case the target antibiotic resistance phenotype was lost, tetracycline resistance in the case of pUTEΔtetM and chloramphenicol resistance in the case of pUTEΔcatQ, generating *B. toyonensis* knockout strains BCT-7112Δtet and BCT-7112Δcat, respectively. The fidelity of the integration event was confirmed by sequencing over the region using primers within the flanking fragments and spectinomycin fragment (Figure 1).

### Construction of strains with additional copies of the *gerIC–nucB* intergenic region

Previous unpublished studies indicated an involvement of the *gerIC–nucB* intergenic region of plasmid pBCT77 as contributing to, or enhancing, the observed tetracycline and chloramphenicol resistances in *B. toyonensis* BCT-7112^T^. The 2003-bp *gerIC–nucB* intergenic region extends from nucleotide 75752–76974 on the pBCT77 genome (NC_022782). In the first place we used primers 1pBCT77Fwd (5′-GCCCCTTTAAACTTCATTATGC-3′) and 4pBCT77Rev (5′-CCCTTACCTTCAAGTATTCC-3′) to confirm that pBCT77 was still present in both knockout strains. The intergenic DNA was then synthesized and cloned into the *Hin*dIII and *Xba*I sites of pUTE853 (DNA 2.0, Newark, California). The resulting vector, pIGR1, was transformed into *E. coli* strain DH5α and its structure confirmed by restriction analysis and PCR. Following passage through the *E. coli dam-*minus strain GM48, pIGR1 was electro-transformed into *B. toyonensis* strains BCT-7112^T^, BCT-7112Δtet, BCT-7112Δcat, and Rock1-3, selecting for erythromycin resistant transformants at 37°C. Erythromycin selection was maintained in all pre-cultures, but not during MIC experiments.

### Construction *B. subtilis* strains with *tetM* and *catQ* genes from BCT-7112^T^, Rock1-3, and Rock3-28

For expression in *B. subtilis*, the *tetM*, and *catQ* genes from BCT-7112^T^, Rock1-3, and Rock3-28 were first cloned into the bifunctional expression/integration vector pDR111 (Britton et al., 2002). This vector has a replication origin for *E. coli* but not *B. subtilis*, and therefore can only persist in *B. subtilis* by integration into the *amyE* locus *via* front-end and back-end fragments of this gene. The intervening sequences include a spectinomycin resistance genes and a copy of *lacI* encoding the Lac repressor. Finally, downstream of a strong P_hyper-spank_ promoter is a multiple cloning site. The respective *tetM* and *catQ* genes were PCR amplified from the *B. toyonensis* strains with *Sal*I and *Sph*I sites using the following primers: (i) for the *tetM* genes of strains BCT-7112^T^ and Rock1-3 strains, tetMFwd1 (5′-GGCCGGGTCGACTGAGCATGAACTTGTCAAACTACCC-3′) and tetMRev1 (5′- GGCCGGGCATGCGTAATAGAAACACTTAAAGAAGTTGTTAGG-3′) and strain Rock3-28, tetMFwd2 (5′- GGCCGGGTCGACTGAGCATGAACTTGTCAAATTACCC-3′) and tetMRev2 (5′- GGCCGG-GCATGC-GCGCCCTCTAGTAAGGAAAAGTATC-3′); (ii) for the catQ genes of strains BCT-7112^T^, Rock1-3, and Rock3-28, catQFwd1 (5′- GGCCGGGTCGACTATTAATAGTATGTGGATGGATTGC-3′) and catQRev1 (5′- GGCCGGGCATGCCAATTTTATATAATTAAAAATTTGATATTACTTAAAGCC-3′). Following ligation and transformation initially into *E. coli* strain DH5α, the structures of the recombinant plasmids (pDRtetM7112, pDRtetM1-3, pDRtetM3-28, pDRcatQ7112, pDRcatQ1-3, and pDRcatQ3-28, see Table 1) were confirmed by digestion and then transformed into *B. subtilis* 168 by natural transformation (Anagnostopoulos and Spizizen, 1961). The resulting strains were: for the *tetM* constructs, *B. subtilis* strains 168tetM7112, 168tetM1-3, and 168tetM3-28; and for the *catQ* constructs, *B. subtilis* strains 168catQ7112, 168catQ1-3, and 168catQ3-28 (Table 1).

The growth parameters of *B. subtilis* with integrated copies of the *tetM* and *catQ* genes from *B. toyonensis* strains BCT-7112^T^, Rock1-3, and Rock3-28 were determined in the presence of various concentrations of tetracycline and chloramphenicol. Antibiotic solutions (0, 4, 8, 16, and 32 μg/ml) were prepared in Brain Heart Infusion broth, prewarmed to 37°C and distributed to in 800 μl amounts into a 48-well FlowerPlate® (m2pLabs, Baesweiler, Germany). The individual wells were inoculated to an OD_600_ of 0.05 with the required cultures (168tetM7112, 168tetM1-3, 168tetM3-28, 168catQ7112, 168catQ1-3, and 168catQ3-28), previously grown overnight in Brain Heart Infusion broth without antibiotics. The experiment was duplicated with one half of the cultures containing IPTG (1 mM) to induced expression from the upstream P_hyper-spanc_ promoter and the other half relying on their cognate upstream regulatory regions. The cultures were incubated in a BioLector® micro bioreactor (m2pLabs) for 20 h at 37°C, with a gain of 25 and shaking at 1500 rpm. The BioLector microreactor determines the optical density of the culture by measuring the scattered light signal using an excitation light beam with a wavelength of 620 nm. The data is shown on a linear rather than logarithmic graph to make it easier to compare growth profiles.

### Visualization of cells by light microscopy

Overnight cultures in Müller Hinton Broth (MHB) were used to inoculate fresh broth to an OD_600_ of 0.05. The cells were grown at 37°C with shaking until the OD_600_ reached 0.2 when chloramphenicol (2 or 32 μg/ml) was added. Samples were taken every 60 min for visualization by phase contrast microscopy.

### Relative quantification of *tetM* and *catQ* genes expression

The expression of the *tetM* and *catQ* genes was determined by a combination of Northern Blotting and quantitative (q) PCR, the former used to establish the transcript sizes the latter for quantitation.

For Northern blotting the Total RNA purification Kit (Norgen Biotek Corp) was used for the extraction of total RNA. The RNA integrity was determined by ensuring the A_260/280_ ratio was between 1.8 and 2.0 and by electrophoresis on a denaturing 1.2% agarose-formaldehyde gel. Northern Blot analysis was performed as described by Homuth et al. (1997). Digoxigenin (DIG) labeled *catQ*- and *tetM*-specific RNA probes were synthesized by *in vitro* transcription using T7 RNA polymerase and a DIG RNA Labelling Kit (Roche Diagnostics) from specific PCR products as templates. Primer sequences used to amplify the templates for the RNA probes were as follows: catQ-FWD: 5′-GTTGATATTACGATGATGCTAGAGAAAATAC-3′, catQ-REV: 5′-CTAATACGACTCACTATAGGGAGACTTTCGGGAAGAGACCATGAAC-3′; tetM-FWD: 5′-ATGAAACGAATGTGATTAAAGAAATTGGC-3′, tetM-REV: 5′-CTAATACGACTCACTATAGGGAGAGGTGCTACTCGTTCCATACAATC-3′. The hybridization signal was developed using a digoxigenin-specific antibody conjugated to alkaline phosphatase (Roche Diagnostics) and CDP-Star (Thermo Fisher Scientific) as a chemiluminescence substrate. The images were captured on an ImageQuant LAS 4000 (GE Healthcare Life Sciences).

In the case of qPCR, the unlabeled PCR primers and TaqMan® MGB probes (5′-FAM™ dye labeled) were designed using the Assays-by-Design™ service (Applied Biosystems). The primers and probes used were as follows: for the *tetM* gene, TETP_Fwd (5′-CGGAAATGCAGTTCAAGCTTCTAC-3′), TETP_Rev (5′-AATTTTAATATCACTCAGTCCCCACACTT-3′), and TETP_probe (5′-CTAGCGGAGAATTTT-3); for the catQ gene, CAT._Fwd (5′-CTAATGTTCATGGTCTCTTCCCGAAA-3′), CAT._Rev 5′-CTAGTCCAAGGTATCCCAGAAATCG-3′), and CAT_probe (5′-TTGGCGGAATATTTTC-3′); for the *gyrB* gene, GYRB_Fwd (5′-CAGCAATCGTGTCAATTAAACATCCAA-3′), GYRB_Rev (5′-CCGTAATCGTTCTCGCTTCACTATT-3′), and GYRB_probe (5′-ACGAAGACGAAACTTG-3′).

The RNAprotect Bacteria Reagent (QIAGEN) was used to stabilize RNA by preventing the degradation of RNA transcripts and the induction of genes. The bacterial strains were cultured in Luria Bertani (LB) medium at 37°C. Depending on the strain and species, the incubation time required to reach exponential phase was 2–3 h before harvesting the cells. RNA extraction was performed using the RNeasy Protect Bacteria MiniKit (QIAGEN), according to the manufacturer's instructions. The expression of the housekeeping gene *gyrB* was used as baseline control for relative quantification.

The quality and concentration of RNA was measured using a Nanodrop 1000 Spectrophotometer, as recommended in the high capacity cDNA reverse transcription kit (Applied Biosystems), and adjusted at 50 ng/μl for relative quantification and PCR normalization. The quantitative PCR was performed using the qPCR MasterMix No ROX (Eurogenetec) and the Custom TaqMan Gene Expression Assays system (Applied Biosystems). The calculation of the relative expression ratios of the target genes was based on the mathematical model of Pfaffl (2001).

### Bioinformatical analyses

BLASTp was used to identify homologs of the *B. toyonensis* 7112^T^ CatQ and TetM proteins among sequences in the NCBI genome database (Altschul et al., 1990). The data was downloaded in CVS format and uploaded into Excel (Tables S1, S2). The data shows the Reference Sequence IDs, individual Sequence IDs, the description of the protein in the annotation file, the bacterial source, alignment score, *E*-value and % identity. The BLASTp data was also used to construct Neighbor Joining trees of homologs of the *B. toyonensis* BCT-7112^T^ CatQ and TetM proteins in the NCBI genome database. The algorithm used (Saitou and Nei, 1987) produces an un-rooted tree. The maximum allowed fraction of mismatched bases between any pair of sequences was 0.85. The evolutionary distance between two sequences was modeled as the expected fraction of amino acid substitutions per site, based on the fraction of mismatched amino acids in the aligned region (Grishin, 1995).

## Results

### Characterization of resistance phenotypes and the identification of resistance genes

Minimum inhibitory concentrations (MICs) of *B. toyonensis* BCT-7112^T^ for tetracycline and chloramphenicol were, at 16 and 32 μg/ml respectively, higher than the resistance break points (≤8 μg/ml) identified by the European Committee on Antimicrobial Susceptibility Testing (Kahlmeter et al., 2006). Consequently *B. toyonensis* BCT-7112^T^ is designated as being resistant to both of these antibiotics. Analysis of the annotated *B. toyonensis* BCT-7112^T^ genome sequence (NCBI; CP006863), and plasmids pBCT77 (CP006864) and pBCT8 (CP006865), revealed the presence of genes encoding putative tetracycline and chloramphenicol resistance proteins.

The most common tetracycline resistance mechanisms are efflux pumps and ribosome-protection proteins (Wilson, 2014). The *B. toyonensis* BCT-7112^T^ genome encodes two putative tetracycline resistance proteins, one an efflux protein and the other a putative ribosome-protection protein. Btoyo_4389 (nucleotides 4295949–4297223) is annotated as encoding a tetracycline resistance protein of the Major Facilitator Superfamily (MFS, cd06174). The MFS is a large and diverse group of secondary transporters that includes uniporters, symporters, and antiporters. MFS proteins facilitate transport across the cytoplasmic membrane of various substrates, including ions, sugar phosphates, antibiotics, amino acids, and peptides. MFS transporters are common among soil-living organisms and, without specific experimental evidence, it can be difficult to identity their substrates. BLAST analysis revealed that homologs of Btoyo_4389 are found extensively among members of the *B. cereus* group but are labeled, in decreasing order of frequency, “MFS transporter,” “tetracycline resistance protein,” major facilitator protein,” and “putative multidrug resistance protein.” It is not clear what, if any, evidence there is for annotating this protein specifically as a “tetracycline resistance protein” rather than as a “MFS transporter,” illustrating a limitation of automated annotation programs.

Btoyo_0322 (nucleotides 307023–308966) is a putative ribosome protection protein with a conserved TetM-like family domain (cd04168). TetM proteins exhibit ~45% similarity with elongation factor G (EF-G) proteins. They have conserved nucleotide-binding motifs and are members of the translation factor superfamily of GTPases that bring about the release of tetracycline from the ribosome in a GTP-dependent manner (Dönhöfer et al., 2012).

Several genes are annotated as encoding a TetR-like protein. TetR proteins are a class of transcriptional regulators with helix-turn-helix motifs, and are so annotated because the first representative of this class was responsible for the regulation of a MFS transporter mediating tetracycline resistance on transposon Tn*10* (Beck et al., 1982). The association with tetracycline is therefore historic.

Analysis of the *B. toyonensis* BCT-7112^T^ genome reveals the presence of two genes encoding putative chloramphenicol acetyltransferases (Cat) that inactivate chloramphenicol by acetylation. Btoyo_3723 (nucleotides 3655712–3656095), at 127 residues in length, is significantly shorter than other chloramphenicol acetyltransferases (~220 residues) and is likely to have been truncated during evolution. Although, the active site is present, it is unlikely that this gene encodes an active enzyme. In contrast, Btoyo_4985 (nucleotides 4933576–4934223) encodes a full-length CatQ-like chloramphenicol acetyltransferase.

A previous unpublished transposon mutagenesis study (EFSA-FEEDAP, 2014; M Kato, Science Tanaka, Japan, personal communication) identified a region on pBCT77, between *nucB* (Btoyo_5072 [nucleotides 74482–74916]) and *gerIC* (Btoyo_4992 [nucleotides 250–858]) that had a minor influence on reducing the MICs of tetracycline and chloramphenicol in *B. toyonensis* BCT-7112^T^. Two of the three genes in this region encode proteins of unknown function (Btoyo_5073 [nucleotides 74939–75190]; Btoyo_5074 [nucleotides 75202–75432]), while the third gene (Btoyo_5075 [nucleotides 75457–76029]) encodes a phage-type site-specific recombinase. There were no genes encoding known antibiotic resistance genes in this region, elsewhere on this pBCT77 or on the smaller plasmid, pBCT8.

### Identification of the genes encoding tetracycline and chloramphenicol resistances

Btoyo_0322 and Btoyo_4389 were tentatively identified as encoding tetracycline resistance proteins, the former a TetM-like protein, the later a MFS-like transporter. Because of limitations in the identification of MFS substrates and the higher likelihood that Btoyo_4389 was incorrectly annotated, we focused on Btoyo_0322. The tetM gene encoded by Btoyo_0322 was replaced with a spectinomycin resistance gene (Section Construction of *tetM* and *catQ* Knockout Strains) and the resulting knockout strain (BCT-7112Δtet) was spectinomycin and chloramphenicol resistant, but erythromycin and tetracycline susceptible. The authenticity of the integration event was confirmed by sequencing across the entire region (see Figure 1A). The data confirmed that Btoyo_0322 was excised from the chromosome and that this gene was solely responsible for the observed tetracycline resistance phenotype of *B. toyonensis* strain BCT-7112^T^.

A similar approach was used to identify the gene responsible for chloramphenicol resistance. Of the two putative chloramphenicol resistance genes identified in the genome of BCT-7112^T^, Btoyo_3723, and Btoyo_4985, the later was chosen as the former appeared to have been truncated. The gene replacement strategy described in Section Construction of *tetM* and *catQ* Knockout Strains was employed, involving the replacement of Btoyo_4985 with a spectinomycin resistance gene. The resulting knockout strain (BCT-7112Δcat), was spectinomycin and tetracycline resistant, but erythromycin and chloramphenicol susceptible. The authenticity of the integration event was again confirmed by sequencing across the entire region (see Figure 1B). The data confirmed that Btoyo_4985 was solely responsible for the observed chloramphenicol resistance phenotype of *B. toyonensis* strain BCT-7112^T^.

Following their identification, the %GC contents of Btoyo_0332 and Btoyo_4985 were determined to be 37.3 and 29.8%, respectively, while the genome average of *B. toyonensis* BCT-7112^T^ was 35.6%. Using a 1000-bp window and a standard deviation cut-off value of 2.5, neither the %GC of these coding sequences, nor a 3500-bp regions either side, were found to be significantly different from the running average, indicating that Btoyo_0332 and Btoyo_4985 are not recent acquisitions.

### The presence of homologs of *tetM* (Btoyo_322) and *catQ* (Btoyo_4985) genes in other member of the *B. cereus sensu lato* group

In light of the recent studies revising the taxonomic status of the *B. cereus* group (*sensu lato*) (Jiménez et al., 2013; Böhm et al., 2015; Liu et al., 2015), we used BLASTp (Altschul et al., 1990) to search for homologs of the BCT-7112^T^ *tetM* and *catQ* genes in the other 18 strains within the *B. toyonensis* taxonomic unit. The data (Table 2) clearly show that homologs of TetM are present in all strains of *B. toyonensis* while homologs of CatQ are present in all but one strain. The failure to detect CatQ in *B. toyonensis* VD115 is most likely to be due to the absence of the relevant region in the partial genome sequence, which is 3884 k in length compare with 4940 k for *B. toyonensis* BCT-7112^T^.

**Table 2:** **The percentage identity and similarity of proteins encoded by homologs of the ***tetM*** and ***catQ*** genes of ***B. toyonensis*** BCT-7112^**T**^ among other members of the ***B. toyonensis*** taxonomic unit**.

**Strain**	**GenBank Accession N^o^**	**% Identity/Similarity to *B. toyonensis* BCT-7112 TetM**	**% Identity/Similarity to *B. toyonensis* BCT-7112 CatQ**
*B. toyonensis* BCT-7112^T^	CP006863.1	100/100	100/100
*B. toyonensis* VD115^*^	JH792165.1	97/98	TS^***^
*B. toyonensis* MC28^*^	CP003687.1	94/97	100/100
*B. toyonensis* Rock3-28^*^	CM000730.1	93/96	100/100
*B. toyonensis* VD131^**^	KB976660.1	93/96	100/100
*B. toyonensis* HuB5-5^**^	JH792120.1	100/100	100/100
*B. toyonensis* VD148^*^	JH792156.1	90/95	100/100
*B. toyonensis* BAG2O-2^**^	AHCX01000000.1	93/96	100/100
*B. toyonensis* Rock1-3^*^	CM000728.1.1	100/100	100/100
*B. toyonensis* BAG1O-2^*^	AHCO00000000.1	100/100	100/100
*B. toyonensis* HuB4-10^**^	AHEE00000000.1	100/100	100/100
*B. toyonensis* BAG5O-1^*^	AHDI00000000.1	99/99	100/100
*B. toyonensis* HuA2-3^**^	AHDW00000000.1	93/97	100/100
*B. toyonensis* BAG6O-1^*^	JH804627.1	99/100	100/100
*B. toyonensis* BAG4X2-1^*^	JH804617.1	100/100	99/99
*B. toyonensis* VD214^**^	AHFN00000000.1	100/100	99/99
*B. toyonensis* HuB2-9^**^	AHED00000000.1	100/100	99/99
*B. toyonensis* Rock3-29^*^	CM000731.1	100/100	99/99
*B. toyonensis* Rock4-18^**^	CM000735.1.1	93/96	99/99

* Clustered to the B. toyonensis group by Jiménez et al. (2013);

** Clustered to the B. toyonensis group by Liu et al. (2015);

*** *TS, truncated sequence detected*.

*In some cases the NCBI Reference for master record for a whole genome shotgun sequence is given*.

In the case of TetM, just under half of the *B. toyonensis* strains encode proteins with identical sequences (Table 2). The most variable TetM protein is encoded by *B. toyonensis* VD148, which shows 90% identity and 95% similarity with that of strain BCT-7112^T^. Twelve of the *B. toyonensis* strains encode CatQ proteins that are identical to that of strain BCT-7112^T^, while the remaining strains exhibit CatQ proteins with 99% identity. Moreover, the *tetM* and *catQ* genes are located in the same genomic neighborhoods in all of the *B. toyonensis* strains. Taken together, the data indicate that the *tetM* and *catQ* genes are ancient genomic components of the *B. toyonensis* taxonomic unit rather than having been acquired recently by horizontal gene transfer.

The NCBI genomes database contains whole genomes, chromosomes, scaffolds and contiguous fragments (contigs) of *B. anthracis, B. bombysepticus, B. cereus sensu stricto, B. mycoides, B. thuringiensis, B. toyonensis, B. weihenstephanensis*, and *B. wiedmannii* (Table 3). In order to extend the homology study to other members of the *B. cereus sensu lacto*, BLASTp was used to search for homologs of the *B. toyonensis* BCT-7112^T^ TetM and CatQ proteins with a similarity score of 85% or greater (Table 4). All of the strains identified as having homologs of these TetM and CatQ proteins were members of the *B. cereus* group (sensu lato): no strains outside this group appeared in the analysis. Homologs of both the TetM and CatQ were found in all eight members of the *B. cereus* group shown in Table 3. Genes encoding these proteins were absent from *B. cytotoxicus* and *B. pseudomycoides*, although this could be due either to the small number of DNA sequences available for these species or to changes in their taxonomic status. A list of sequences analyzed for homology with the TetM and CatQ proteins is given in Tables S1, S2, respectively.

**Table 3:** **Breakdown of the genomic sequences analyzed to identify homologs of the ***B. toyonensis*** BCT-7112^**T**^ TetM and CatQ proteins**.

**Taxonomic group/ID**	**Complete**	**Chromosome**	**Scaffold**	**Contig**	**Total**
*B. anthracis*/1392	44	10	41	42	137
*B. bombysepticus*/1330043	1	0	0	0	1
*B. cereus (sensu stricto)*/1396	32	29	154	51	266
*B. mycoides*/1405	2	3	3	5	13
*B. thuringiensis*/1428	37	16	14	12	79
*B. toyonensis*/155322	1	0	0	0	1
*B. weihenstephanensis*/86662	2	0	0	4	6
*B. wiedmannii*/1890302	0	0	0	11	11

**Table 4:** **The distribution of proteins encoded by homologs (>85% similarity) of the ***tetM*** and ***catQ*** genes of ***B. toyonensis*** BCT-7112^**T**^ among other members of the ***B. cereus sensu lato*** group**.

**Gene**	***B. thuringiensis***	***B. cereus (sensu stricto)***	***B. anthracis***	***B. mycoides***	***B. weihen-stephanensis***	***B. toyonensis***	***B. wiedmannii***	***B. bombysepticus***	**Total**
CatQ	66	161	111	7	6	18	11	1	381
TetM	77	135	112	4	10	19	11	1	369

A total of 369 homologs of TetM were identified in the NBCI database and widely distributed among species of the *B. cereus* group, broadly reflecting the frequency of complete genomes and chromosomes in the database (Table 3). In the case of CatQ, 381 homologs were found, and with a distribution that was similar to that of TetM. The relationships between these TetM and CatQ sequences, in the form of Neighbor Joining trees, are shown in Figures S1, S2. In both cases the trees exclusively contain homologous proteins from other members of the *B. cereus* group (*sensu lato*), confirming the ancient origin of these proteins in this taxonomic clade.

### Tetracycline and chloramphenicol resistance profiles of the strains within the *B. toyonensis* taxonomic unit

*B. toyonensis* BCT-7112^T^ is phenotypically resistant to tetracycline and chloramphenicol, and this study identified the genes responsible, namely *tetM* (Btoyo_0322) *and catQ* (Btoyo_4985). However, eight other *B. toyonensis* strains are phenotypically susceptible to tetracycline and chloramphenicol, despite encoding TetM and CatQ proteins that are homologous to those strain BCT-7112^T^ (EFSA-FEEDAP, 2014; L. Mocé, Eurofins Biolab, personal communication). We therefore addressed the question of why other members of the *B. toyonensis* taxonomic unit that encode either identical (strain Rock1-3) or similar (strain Rock3-28) resistance determinants do not exhibit the same resistance phenotypes. Both strains are susceptible to tetracycline and chloramphenicol (MIC 1 μg/ml) and have similar growth kinetics and therefore only the growth profiles of strain Rock1-3 are shown in Figure S3, in comparison with the profiles of strains BCT-7112^T^, BCT-7112Δtet, and BCT-7112Δcat.

When *B. toyonensis* BCT-7112^T^ was grown in the presence of various concentrations of tetracycline (0, 1, 2, 4, 16, and 32 μg/ml) the MIC was 16 μg/ml (Figure S3A). When the isogenic strain (BCT-7112Δtet) in which the *tetM* (Btoyo_0322) gene had been replaced with a spectinomycin resistance gene was tested, the MIC was 1 μg/ml (Figure S3B). In contrast, when the isogenic strain (BCT-7112Δcat) in which the *catQ* gene was replaced with a spectinomycin resistance gene, the MIC for tetracycline remained at 16 μg/ml (Figure S3C). In the case of the Rock1-3 strain, there was some initial growth at 1 μg/ml (Figure S3D), but the cells eventually died and the biomass concentration declined, indicating it had an MIC for tetracycline of 1 μg/ml.

Similar growth experiments were performed to determine the MICs for chloramphenicol of *B. toyonensis* strains BCT-7112^T^, BCT-7112Δtet, BCT-7112Δcat, and Rock1-3 (Figures S3E–H). These confirmed that *catQ* was responsible for the chloramphenicol resistance phenotype and that the Rock1-3 strain, with an MIC of 1 μg/ml, was susceptible to this antibiotic.

Previous studies indicated an involvement of the *gerIC–nucB* intergenic region of plasmid pBCT77 in contributing to, or enhancing, the observed resistance of *B. toyonensis* BCT-7112^T^ to tetracycline and chloramphenicol (EFSA-FEEDAP, 2014; Matsumoto, Asahi Vet Japan, personal communication). We therefore confirmed the presence of the *gerIC–nucB* intergenic region in *B. toyonensis* strains BCT-7112^T^, BCT-7112Δtet, and BCT-7112Δcat and also generated versions of these strains with additional copies of this region on plasmid pIGR19 (Section Construction of Strains with Additional Copies of the *gerIC–nucB* Intergenic Region). In addition, a strain of *B. toyonensis* Rock1-3 was generated with additional copies of the intergenic region on pIGR1. The resulting strains were grown in the presence of various concentrations of tetracycline (Figures S4A–D) and chloramphenicol (Figures S4E–G) to determine the influence of the intergenic region on growth and resistance. The data (Figure S4) indicated that the presence of additional copies of the *gerIC*–*nucB* locus do not significantly influence the growth profiles of *B. toyonensis* strains BCT-7112^T^, BCT-7112Δtet, and BCT-7112Δcat on either of the antibiotics. In the case of strain Rock1-3, which encodes TetM and CatQ proteins that are identical to those in strain BCT-7112^T^, but which is susceptible to tetracycline and chloramphenicol, the presence of the *gerIC–nucB* intergenic region had no impact it's sensitivity to these antibiotics. Taken together, these data rule out any impact of the *gerIC–nucB* intergenic region of pBCT77 as a factor in the resistances of *B. toyonensis* BCT-7112^T^ to these antibiotics.

### Why are strains encoding TetM and CatQ susceptible to tetracycline and chloramphenicol?

**Figure 2:**
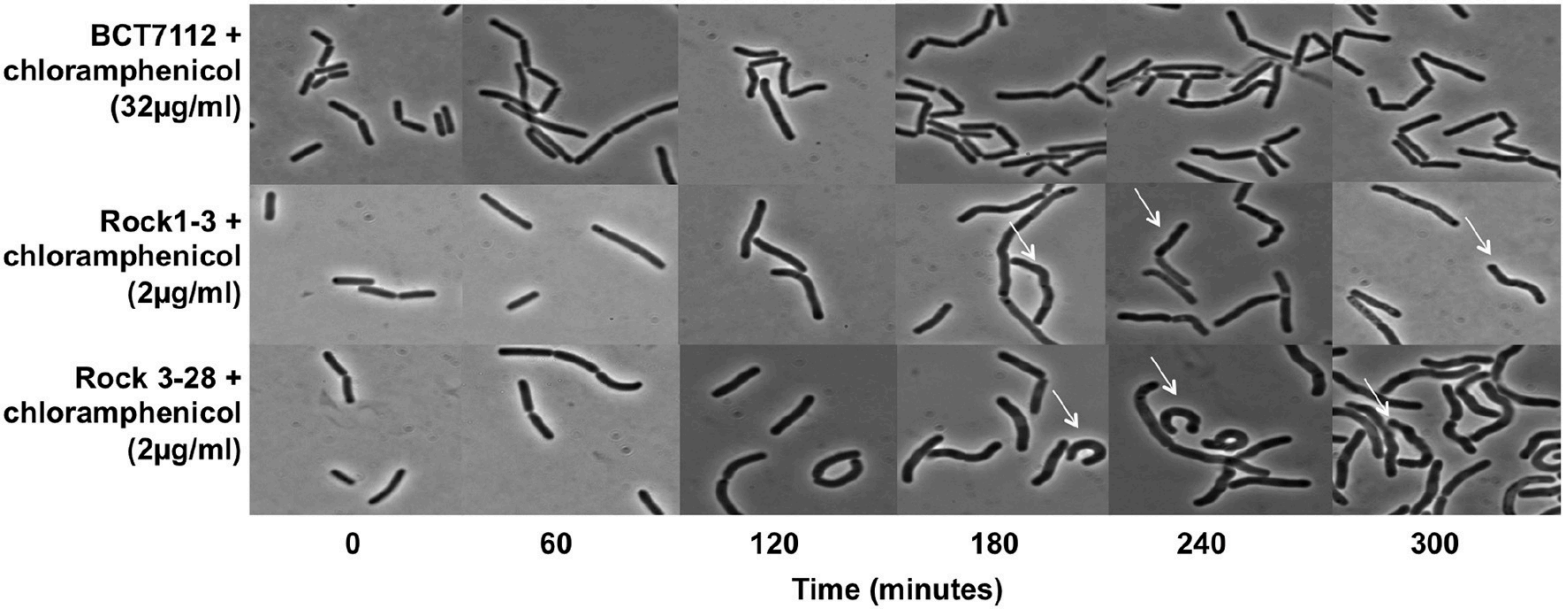
**Influence of chloramphenicol on cell morphology**. *B. toyonensis* strains BCT-7112^T^, Rock1-3, and Rock3-28 were grown in LB medium at concentrations of chloramphenicol just above their MICs. Samples were taken every 60 min and viewed by phase-contrast microscopy. In the case of the Rock1-3 and Rock3-28, twisted cells can be seen in the samples taken at 180 min and later (white arrows).

*B. toyonensis* strains Rock1-3 and Rock3-28 encode identical or almost identical TetM and CatQ proteins to those encoded by strain BCT-7112^T^ (Table 2), but both are susceptible to tetracycline and chloramphenicol (MICs 1 μg/ml). We observed that strains Rock1-3 and Rock3-28 tended to aggregate at or close to their MIC and therefore monitored their morphologies while growing in LB medium in the presence of 2 μg/ml of the antibiotics, comparing them with the morphologies of strain BCT-7112^T^ growing in the presence of the antibiotics with either 2 and 32 μg/ml. Samples were taken every 60 min for microscopy and a representative set of data for growth in the presence of chloramphenicol is shown in Figure 2. During the first 60 min all of the cells appeared normal. However, from ~120 min the morphologies of the Rock1-3 and Rock3-28 strains exhibited distorted cell morphologies compared with BCT-7112^T^ (Figure 2). The observed aggregation of the Rock1-3 and Rock3-28 strains is therefore likely due to this disturbed morphology that ultimately results in cell death.

**Figure 3:**
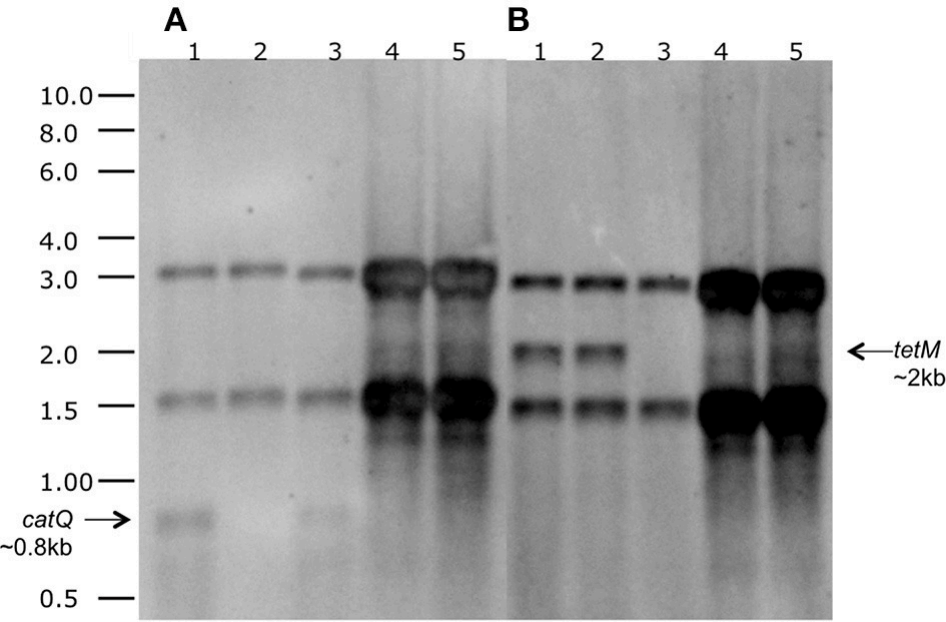
**Northern blot analysis for the detection of ***catQ*** and ***tetM*** transcripts in strains of ***B. toyonensis***. (A)** *catQ-*specific probe; **(B)** *tetM*-specific probe. The tracks contained total RNA from the following strains: 1. BCT-7112^T^; 2. BCT-7112Δcat; 2. BCT-7112Δtet; 4. Rock1–3; 5. Rock3–28. Approximately 5 times more RNA was added to the tracks containing Rock1-3 and Rock3-28 to improve sensitivity. The two prominent bands present in all the tracks are the 30S rRNA (upper) and 16S rRNA (lower).

We next addressed the question of whether the *tetM* and *catQ* genes in strains Rock1-3 and Rock3-28 were expressed. To do this we carried out both Northern blot and qPCR analyses. Northern blotting analysis was carried out on *B. toyonensis* strains BCT-7112^T^, BCT-7112Δcat, BCT-7112Δtet, Rock1-3, and Rock3-28. Cultures were grown in LB to mid-exponential phase and total RNA extracted and electrophoresed on two identical denaturing (1% formalin) agarose gels; to increase sensitivity, ~5 times more RNA was added to the tracks of strains Rock1-3 and Rock3-28 compared with that of strain BCT-7112^T^. Following, hybridization with a DIG-labeled *catQ* probe, monocistronic *catQ*-specific transcripts (~0.8-kb) were detected in strains BCT-7112^T^ and BCT-7112Δtet, but not the other strains (Figure 3A). In the case of the *tetM-*specific probe, strong monocistronic *tetM*-specific transcripts (~2.0-kb) were detected in strains BCT-7112^T^ and BCT-7112Δcat, and weak transcripts in strains Rock1-3 and Rock3-28 (Figure 3B). As expected a *catQ*-specific transcript was absent from strain BCT-7112Δcat and a *tetM*-specific transcript from strain BCT-7112Δtet.

The Northern blot expression data were confirmed by qPCR using *gyrB* as the baseline control. *B. cereus* ATCC 14579 and *B. thuringiensis* ATCC 10792 were included as both encode homologs of the TetM, while *B. thuringiensis* ATCC 10792 only encodes a copy of CatQ. The qPCR data (Table 5) show expression of *tetM* in BCT-7112^T^ and BCT-7112Δcat, but considerably lower or no expression in Rock1-3, BCT-7112Δtet, ATCC 14579, and ATCC 10792. In the case of the *catQ* qRNA data, expression was detected in BCT-7112^T^, BCT-7112Δtet, but again, little or no expression of this gene in BCT-7112Δcat, Rock1-3, ATCC 14579, and ATCC 10792 (Table 5).

**Table 5:** **Quantitative PCR analysis of ***tetM*** and ***catQ*** transcripts in ***B. toyonensis*** strains BCT-7112^**T**^, BCT-7112Δcat, BCT-7112Δtet, and Rock1-3, ***B. cereus*** ATCC 14579 and ***B. thuringiensis*** ATCC 10792**.

**Transcript**	**Strain**	***R* = 2^Δct^**
***tetM***		
	*B. toyonensis* BCT-7112^T^	9.80
	*B. toyonensis* BCT-7112Δcat	5.91
	*B. toyonensis* BCT-7112Δtet	0.0
	*B. toyonensis* Rock1-3	0.18
	*B. cereus* ATCC 14579	0.59
	*B. thuringiensis* ATCC 10792	0.0
***catQ***		
	*B. toyonensis* BCT-7112^T^	233.95
	*B. toyonensis* BCT-7112Δcat	0.13
	*B. toyonensis* BCT-7112Δtet	1706.9
	*B. toyonensis* Rock1-3	3.56
	*B. cereus* ATCC 14579	0.71
	*B. thuringiensis* ATCC 10792	0.01

**Figure 4:**
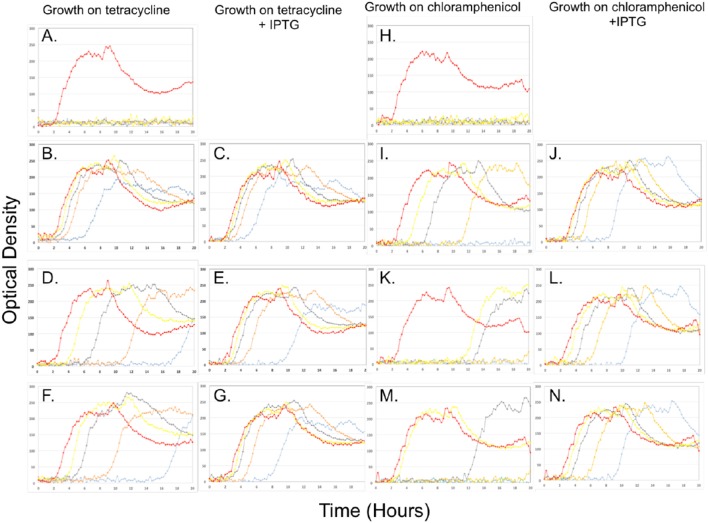
**Growth of ***B. subtilis*** strain 168 (A,H)**, with *tetM* **(A–G)** or *catQ* **(H–N)** genes from BCT-7112^T^ **(B,C,I,J)**, Rock1-3 **(D,E,K,L)** and Rock3-28 **(F,G,M,N)** on various concentrations of tetracycline **(A–G)** or chloramphenicol **(H–N)** with **(C,E,G,J,L,N)** or without **(A,B,D,F,H,I,K,M)** the addition of IPTG (1 mM). The cultures were grown in a BioLector microreactor that determines optical density by measuring the scattered light signal (see Section Visualization of Cells by Light Microscopy). The data is shown on a linear rather than logarithmic graph to make it easier to compare growth profiles. The antibiotic concentrations used were (μg/ml): 0, red; 4, yellow; 8, gray; 16, orange and 32, light blue).

Since *B. toyonensis* strains Rock1-3 and Rock3-28 each contained homologs of the BCT-7112^T^ TetM and CatQ proteins with a high level of identity (Table 2), we carried out experiments to determine if their cognate genes were expressed in the related bacterium *B. subtilis* strain 168. The strategy involved cloning their coding sequences, together with upstream regulatory sequences, into the *B. subtilis* integration/expression vector pDR111. The genes and upstream sequences from *B. toyonensis* BCT-7112^T^ were also cloned as a control. In each case the *tetM* and *catQ* sequences were cloned downstream of the IPTG inducible P_hyper-spank_ promoter in pDR111 and the resulting expression cassette was integrated at the non-essential *amyE* locus of *B. subtilis*. The resulting constructs allow for the expression of the cognate genes from their native promoters or, following IPTG induction, from the P_hyper-spank_ promoter. The growth profiles of the *B. subtilis* strains grown in LB medium with various concentrations of tetracycline or chloramphenicol (0, 4, 8, 16, and 32 μg/ml) and with or without IPTG are shown in Figure 4.

*B. subtilis* is unable to grow in the presence of 4 μg/ml of tetracycline (Figure 4A). Cloning the *tetM* gene from *B. toyonensis* BCT-7112^T^ into *B. subtilis* 168 allows this strain to grow in the presence of 32 μg/ml, albeit with an extended lag phase (Figure 4B). The addition of IPTG to the culture medium did not have a significant influence of growth up to 32 μg/ml tetracycline, indicating that the upstream regulatory region of *tetM* was fully functional in this bacterium (Figure 4B). The growth kinetics for the *B. subtilis* strains encoding the *tetM* genes from strains Rock1-3 (Figures 4C,D) and Rock3-28 (Figures 4F,G) were similar and are discussed together. The data show that, in the absence of IPTG, the *B. subtilis* strains encoding the *tetM* genes from these strains grew in the presence of 32 μg/ml tetracycline. However, the growth kinetics show that the lag phase increased in length with increasing concentrations of tetracycline, to the extent that growth at 32 μg/ml tetracycline is only observed after approximately 18 h. When IPTG is added, the cultures grew without a lag phase up to 8 μg/ml tetracycline and with considerably shorter lag phases at 16 and 32 μg/ml tetracycline. Taken together, the data confirm that the *tetM* genes in *B. toyonensis* strains Rock1-3 and Rock3-28 encode functional proteins, but that expression from their native promoters is weak.

Similar results were observed when *B. subtilis* encodes the *catQ* genes from *B. toyonensis* strains BCT-7112^T^, Rock1-3, and Rock3-28 following growth on chloramphenicol (0, 4, 8, 16, and 32 μg/ml). While *B. subtilis* alone is unable to grow in the presence of 4 μg/ml of chloramphenicol (Figure 4H), the inclusion of the *catQ* gene from strain BCT-7112^T^, together with its native promoter, facilitated growth up to 16 μg/ml (Figure 4I). However, with increasing concentrations of chloramphenicol, there was a longer lag before the culture started to grow, indicating that the native *catQ* promoter was relatively efficient in *B. subtilis*. This was confirmed when IPTG was added to the culture; the lag phases were considerably reduced and the culture grew at 32 μg/ml (Figure 4J). The growth kinetics for the *B. subtilis* strains encoding the *catQ* genes from *B. toyonensis* strains Rock1-3 (Figures 4K,L) and Rock3-28 (Figures 4M,N) were similar with their native promoters facilitating growth at concentrations up to 8 μg/ml, albeit with lag phases different length (Figures 4K,M). When IPTG was included in the culture medium, the cells were able to grow in the presence of 32 μg/ml, but with longer lag phases at the higher antibiotic concentrations (Figures 4L,N).

**Figure 5:**
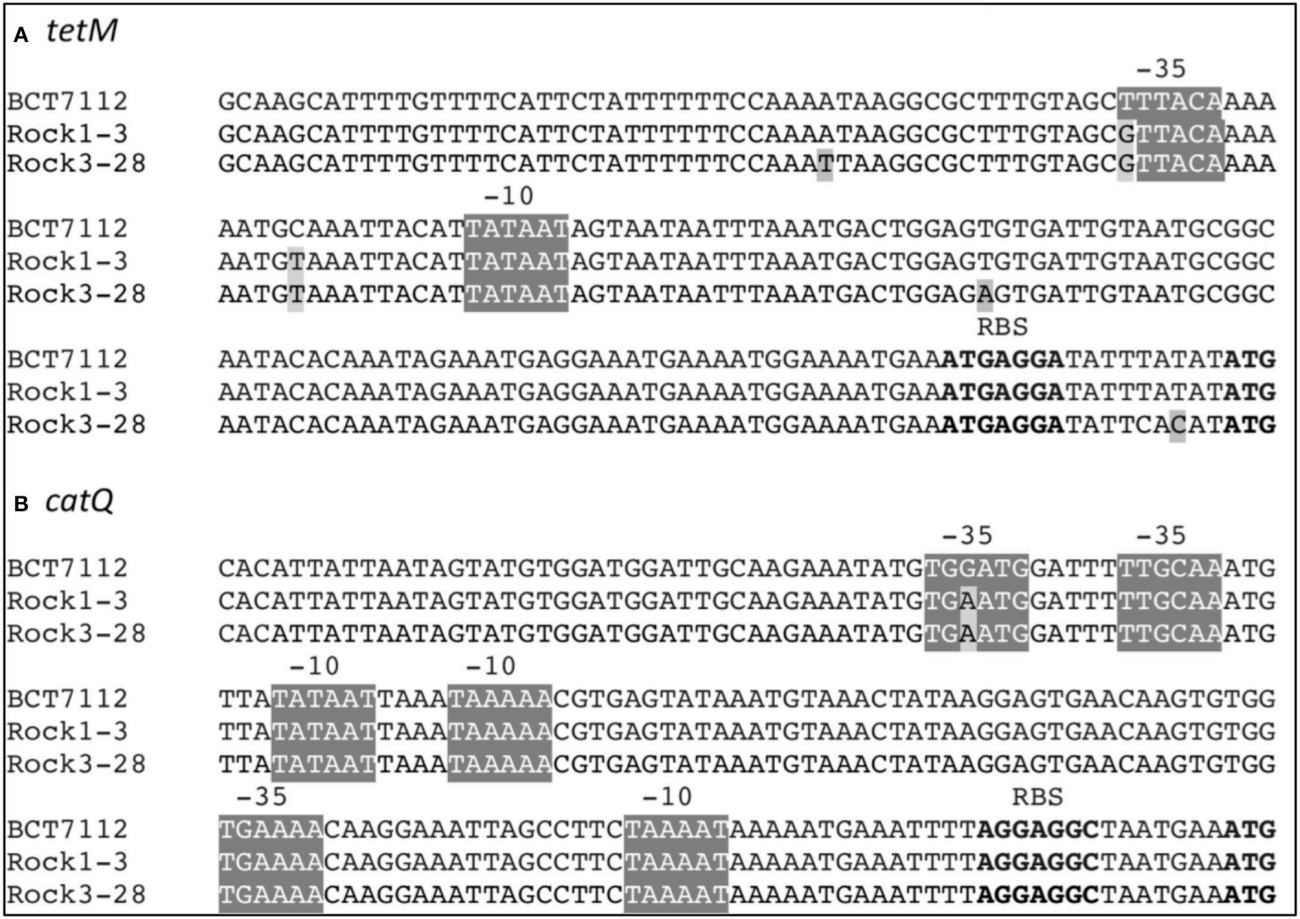
**Nucleotide sequences upstream of the (A)** *tetM* and **(B)** *catQ* genes of *B. toyonensis* strains BCT-7112^T^, Rock1-3, and Rock3-28. Putative Sigma A (vegetative) promoter consensus sequences are shown highlighted (−35/−10) and the ribosome binding sites (RBS) and start codons are in bold. Single nucleotide polymorphisms (SNPs) in the Rock1-3 and Rock3-28 strains are also highlighted.

Taken together, the data in Figure 4 clearly show that the *tetM* and *catQ* genes of *B. toyonensis* strains Rock1-3 and Rock3-28 encode function proteins, capable of conferring resistance to their cognate antibiotics. The data also suggests an explanation for why *B. toyonensis* strain BCT-7112^T^ exhibits resistance to these antibiotics, while strains Rock1-3 and Rock3-28 do not (Figure S3), namely that their upstream promoters are relatively inefficient. Alignment of the nucleotides upstream of *tetM* and *catQ* coding sequences indicates a high degree of identity between the regulatory regions of these genes from strains BCT-7112^T^, Rock1-3, and Rock3-28 (Figure 5). Figure 5 also identifies consensus sequences for putative Sigma A (vegetative) promoters and single nucleotide polymorphisms (SNPs) with respect to BCT-7112^T^. In the case of *tetM*, identical SNPs occur within the putative −35 sequences and within the sequences between the −35 and −10 sequences in Rock1-3 and Rock3-28, with the latter having three additional SNPs in this region (Figure 5A). In the region upstream of *catQ*, three putative Sigma A promoters were identified, the most peripheral of which contains the only SNP observed in this region (Figure 5B).

## Discussion

*B. toyonensis* BCT-7112^T^ is resistant to tetracycline and chloramphenicol and the annotated genome and plasmid sequences were used to identify the putative genes involved. Reciprocal recombination was then used to replace the target resistance genes with a spectinomycin resistance gene and this unambiguously identified Btoyo_0332 (*tetM*) and Btoyo_4985 (*catQ*) as being the genes responsible for the resistance phenotypes in *B. toyonensis* BCT-7112^T^.

We addressed the question of whether the genes responsible for tetracycline and chloramphenicol resistances in BCT-7112^T^ were intrinsic or acquire in three ways. Firstly we established that these genes were not associated with known MGEs (e.g., transposon, transposase, insertion sequence, conjugation etc.). Secondly, we analyzed their %GC content in relation to the genome as a whole and showed that they were not significantly different. Thirdly, we showed that homologous genes were not only present in closely related species, but were also present in virtually all representative of the *B. cereus* group *sensu lato*.

The data (Tables 2, 4) clearly show that the *tetM* and *catQ* genes are widely distributed among members of the *B. cereus* group *sensu lato*, are located within the same genomic neighborhoods and are therefore unambiguously of ancient origin within this group, rather than having been acquired as the result of more recent horizontal gene transfer. This conclusion is supported by animal feeding experiments in which qPCR was used to quantify tetracycline and chloramphenicol resistance genes in intestinal samples from piglets and cattle (Casanovas-Massana et al., 2014). The conclusion from these studies is that the presence of *B. toyonensis* does not contribute significantly to the antibiotic resistance load already present in the intestinal tract of these animals.

While *B. toyonensis* BCT-7112^T^ is resistant to tetracycline and chloramphenicol, closely related strains (e.g., Rock1-3 and Rock3-28) with identical or virtually identical genes and upstream regulatory regions are susceptible to these antibiotics. To address this apparent contradiction, we sought answers to two specific questions: are the cognate resistance genes in Rock1-3 and Rock3-28 functional and, if so, why do they not elaborate the expected resistance phenotype? Using Rock1-3 and Rock3-28 as representative strains, we integrated their *tetM* and *catQ* genes, together with their upstream regulatory sequences, into the *amyE* locus of *B. subtilis*. The data (Figure 4) show firstly that the products of these genes are functionally active since induction of an upstream P_hyper-spank_ promoter conferred the cognate resistance on the *B. subtilis* host. More significantly, when using their native regulatory sequences, these genes were able to confer resistance to *B. subtilis* at levels above its MIC (~1 μg/ml), albeit with varying degrees of efficiency and extended lag phases. This suggests that the native promoters of these genes are relatively weak, and this was confirmed by analysis of their transcript levels (Figure 3 and Table 5). Analysis of cells taken from the non-induced cultures of *B. toyonensis* Rock1-3 growing on tetracycline (16 μg/ml, Figure 4D) and chloramphenicol (8 μg/ml, Figure 4K) revealed that the majority of the cells had become resistant to the cognate antibiotic, presumably by the selection of cells within the population with spontaneous mutations in either the native upstream regulatory sequences or elements of the P_hyper-spank_ promoter system. It is likely that the extended lag phases in these cultures reflect the time taken for such mutants to accumulate in the population.

The final question we addressed is why the *B. cereus sensu lato* group encodes genes that are so poorly expressed that they do not register a phenotype in the standard protocols used for regulatory purposes to identify MIC values. Although, we cannot provide a definitive answer this question, their maintenance over such a long evolutionary time-scale suggests that they must have a specific role or selective advantage in their natural environment, the soil. One possibility is that they are transcribed from non-vegetative Sigma factor promoters induced, for example, in response to specific stresses, quorum sensing pheromones or differentiation processes, and that are not encountered under the MIC test conditions. Another is that their expression has been down regulated over evolutionary time by single base-pair mutations that, as a bet-hedging strategy (Ferenci and Maharjan, 2015), are able to revert at a high frequency and subsequently selected under appropriate conditions. This might account for the resistance phenotypes of *B. toyonensis* BCT-7112^T^.

The results represent a potential issue for regulatory authorities who tend to rely on phenotypic data for assessing the implications of introducing antibiotic resistance organisms into the food chain. For example, in their conclusions on the antibiotic susceptibility of *B. toyonensis* NCIMB 14858^T^ (i.e., BCT-7112T) the EFSA FEEDAP Panel stated the following: “*Although, there is some evidence of the presence of the catQ and tet(M) genes in eight closely related strains, none of these demonstrated the resistant phenotype. Consequently, resistance to chloramphenicol and tetracycline cannot be considered intrinsic to the newly defined species*” (EFSA-FEEDAP, 2014). This statement raises the important issue of exactly what evidence regulatory authorities should consider before concluding whether or not a resistance factor (genotype or phenotype) is intrinsic. The traditional reliance on phenotype is clearly insufficient in the post-genomic era since the intrinsic nature of an observed phenotype can only be defined at the level of its gene. This is particularly the case with resistance determinants since the same phenotype can be encoded by entirely different mechanisms (Blair et al., 2015). Moreover, detecting homologous genes in both closely related and distantly related organisms, particularly if located at similar genomic neighborhoods, strengthens the case for recognizing that the gene, rather than it observable phenotype, is intrinsic.

The question remains as to whether or not strains that are intrinsically resistant to antibiotic(s) should be used in any process associated with the human food chain? It is likely that antibiotics (also referred to as secondary metabolites) have been produced for over 500 million years, dating back to the Cambrian period (Baltz, 2008; Cox and Wright, 2013). Their production relates to evolutionary processes taking place millions of years before the use of antimicrobial chemotherapy, when the antibiotics encountered by bacteria were produced by competitor organisms in the same environment (Perry and Wright, 2013). In some cases, the evolution of impermeability barriers such as the outer membrane, conferred resistance (e.g., erythromycin resistance in *Escherichia coli*), in others existing genes were duplicated and modified (e.g., the elongation factor TU in the case if TetM) or were acquired from the producers themselves (e.g., MFS efflux pumps). It would be unrealistic to exclude the use of all such strains. A realistic approach requires a detailed analysis that: (i) identifies the gene(s) responsible for the resistance phenotype, (ii), confirms that they are not associated with known MGEs (e.g., plasmids, prophages, transposons, insertion sequence, resistance cassettes, integrons etc.), and (iii) irrespective of phenotype, that homologous genes are present in related strains/species and at similar genomic locations.

In conclusion our data clearly show that the *tetM* and *catQ* genes of *B. toyonensis* BCT-7112^T^ are intrinsic not only to this species, but also to the *B*. cereus group (*sensu lato*). They are confirmed to be of ancient origin, since genes subject to horizontal gene transfer would be associated with MGEs, have a significantly different %GC and located at a variety of genomic neighborhoods. The proven intrinsic nature and non-transferability of these antibiotic resistance genes in *B. toyonensis* BCT-7112^T^ counts in favor of recommending the safe use of this strain as an additive in animal nutrition. These studies also show that intrinsic resistance should be defined at the genomic rather than the phenotypic level and this should be taken into account whenever a scientific assessment of the nature and genetic basis of a bacterial antibiotic resistance is performed.

## Author contributions

All authors were involved in the design of the studies and in the interpretation of the data. HG and SP carried out the majority of the experimental work at Newcastle University, under the supervision of CH. FN and EM carried out the qPCR experiments at the Hospital de la Santa Creu i Sant Pau. In addition to their involvement in the design of the studies, GJ and AB provided background information, strains and a critical input into the preparation of the manuscript.

### Conflict of interest statement

The research was funded by Rubinum S.A. that has a commercial interest in *B. toyonensis* strain BCT-7112^T^. However, the other authors declare that the research was conducted in the absence of any commercial or financial relationships that could be construed as a potential conflict of interest. The reviewer AJ declared a shared affiliation, though no other collaboration, with one of the authors AB to the handling Editor, who ensured that the process nevertheless met the standards of a fair and objective review.

## Supplementary material

Table S1 **BLASTp was used to identify homologs of the ***B. toyonensis*** 7112^**T**^ TetM protein against sequences in the NCBI genome database (Altschul et al., 1990)**. The data was downloaded in CVS format and uploaded into Excel. The data shows the Reference Sequence IDs, individual Sequence IDs, the description of the protein in the annotation file, the bacterial source, alignment score, *E-*value and % identity.

Table S2 **BLASTp was used to identify homologs of the ***B. toyonensis*** 7112^**T**^ CatQ protein against sequences in the NCBI genome database (Altschul et al., 1990)**. The data was downloaded in CVS format and uploaded into Excel. The data shows the Reference Sequence IDs, individual Sequence IDs, the description of the protein in the annotation file, the bacterial source, alignment score, *E*-value and % identity.

Figure S1 **Neighbor Joining tree of homologs of the ***B. toyonensis*** BCT-7112^**T**^ TetM tetracycline resistance protein (Btoyo_0322) identified in the BLASTp analysis**. The algorithm used produces an un-rooted tree (Saitou and Nei, 1987). The maximum allowed fraction of mismatched bases between any pair of sequences was 0.85. The evolutionary distance between two sequences was modeled as the expected fraction of amino acid substitutions per site, based on the fraction of mismatched amino acids in the aligned region (Grishin, 1995).

Figure S2 **Neighbor Joining tree of the homologs of the ***B. toyonensis*** BCT-7112^**T**^ CatQ chloramphenicol resistance protein (Btoyo_4985) identified in the BLASTp analysis**. The algorithm used produces an un-rooted tree (Saitou and Nei, 1987). The maximum allowed fraction of mismatched bases between any pair of sequences was 0.85. The evolutionary distance between two sequences was modeled as the expected fraction of amino acid substitutions per site, based on the fraction of mismatched amino acids in the aligned region (Grishin, 1995).

Figure S3 **Minimum inhibitory concentrations (MICs) of strains of ***B. toyonensis*** on tetracycline (A–D)** and chloramphenicol **(E–H). (A/E)**, BCT-7112^T^; **(B/F)**, BCT-7112Δtet; **(C/G)**, BCT-7112Δcat; **(D/H)** Rock1-3. The antibiotic concentrations used were (μg/ml): 0, red; 1, green; 2, dark blue; 4, yellow; 8, gray; 16, orange and 32, light blue. The MIC values are shown for each set of growth curves. The experiments were carried out a minimum of three times and representative data show on a linear rather than logarithmic graph to make it easier to compare growth profiles.

Figure S4 **The impact of the ***gerIC–nucB*** intergenic region of plasmid pBCT77 on the tetracycline (A–D)** and chloramphenicol **(E–H)** resistance profiles of the following strains: **(A/E)**, BCT-7112(pIGR1); **(B/F)**, BCT-7112Δtet(pIGR1); **(C/G)**, BCT-7112Δcat(pIGR1); **(D/H)**, Rock1-3(pIGR1). The antibiotic concentrations used were (μg/ml): 0, red; 1, green; 2, dark blue; 4, yellow; 8, gray; 16, orange and 32, light blue. The MIC values are shown for each set of growth curves. The experiments were carried out a minimum of three times and representative data show on a linear rather than logarithmic graph to make it easier to compare growth profiles.
