# Supplementary figures and images for: The Identification of Intrinsic Chloramphenicol and Tetracycline Resistance Genes in Members of the Bacillus cereus Group (sensu lato)
Source: Front Microbiol. 2017 Jan 4;7:2122. doi: 10.3389/fmicb.2016.02122 (PMC5209696; doi:10.3389/fmicb.2016.02122)

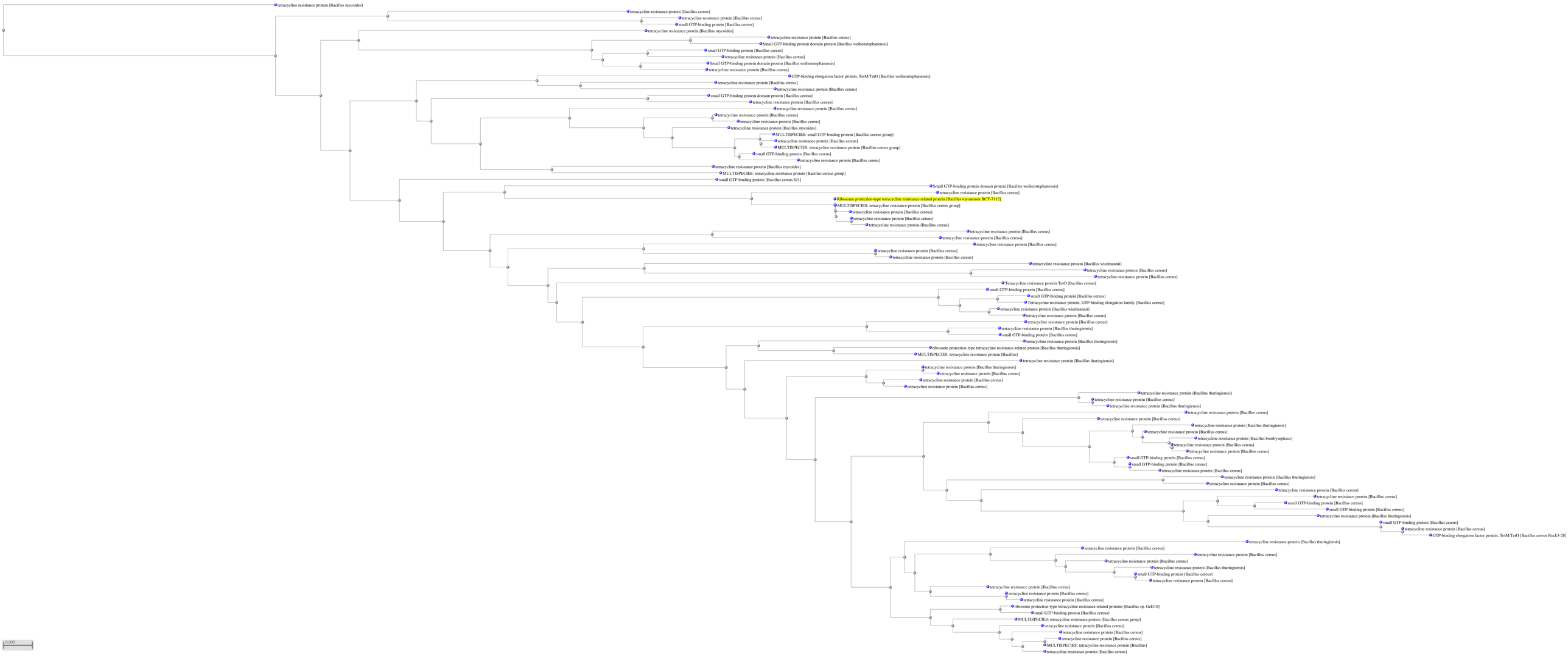

Supplement: Figure S1 — Neighbor Joining tree of homologs of the B. toyonensis BCT-7112T TetM tetracycline resistance protein (Btoyo_0322) identified in the BLASTp analysis. The algorithm used produces an un-rooted tree (Saitou and Nei, 1987). The maximum allowed fraction of mismatched bases between any pair of sequences was 0.85. The evolutionary distance between two sequences was modeled as the expected fraction of amino acid substitutions per site, based on the fraction of mismatched amino acids in the aligned region (Grishin, 1995). [file Image1.PDF]

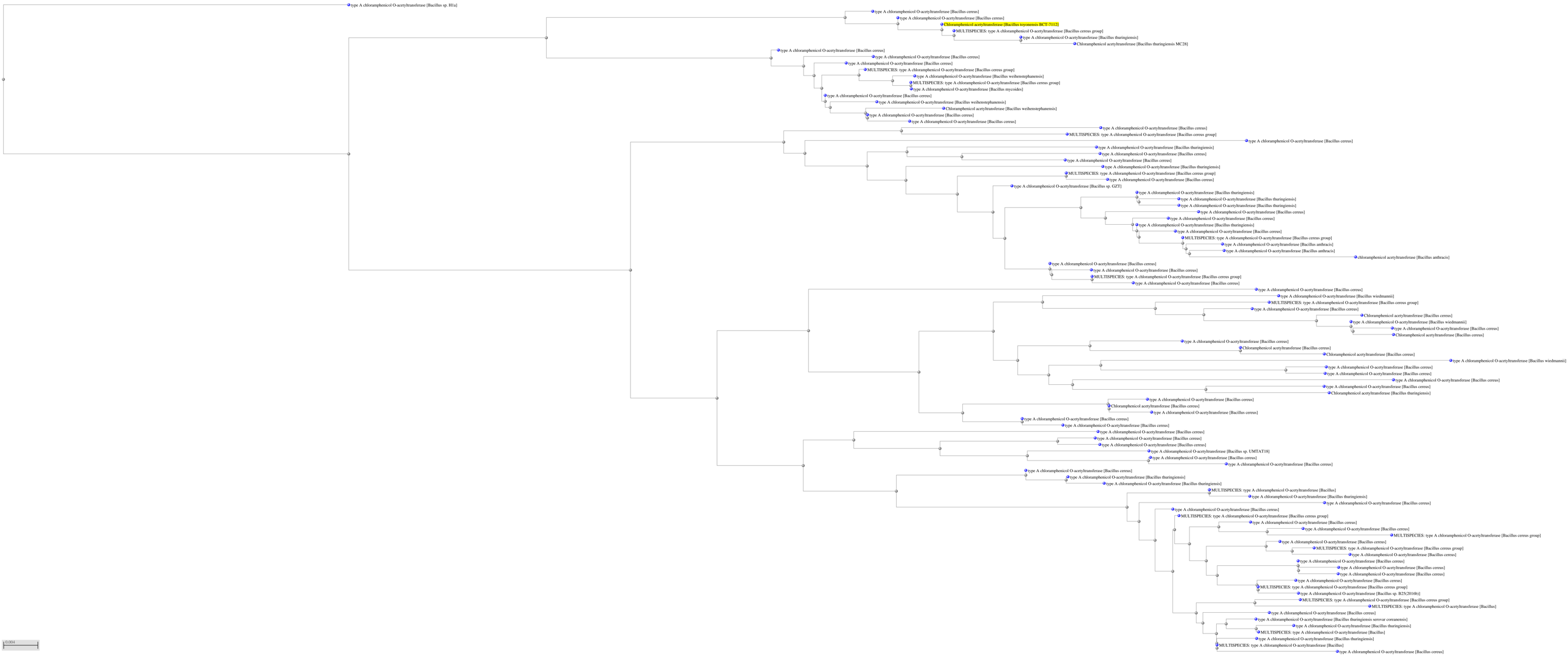

Supplement: Figure S2 — Neighbor Joining tree of the homologs of the B. toyonensis BCT-7112T CatQ chloramphenicol resistance protein (Btoyo_4985) identified in the BLASTp analysis. The algorithm used produces an un-rooted tree (Saitou and Nei, 1987). The maximum allowed fraction of mismatched bases between any pair of sequences was 0.85. The evolutionary distance between two sequences was modeled as the expected fraction of amino acid substitutions per site, based on the fraction of mismatched amino acids in the aligned region (Grishin, 1995). [file Image2.PDF]

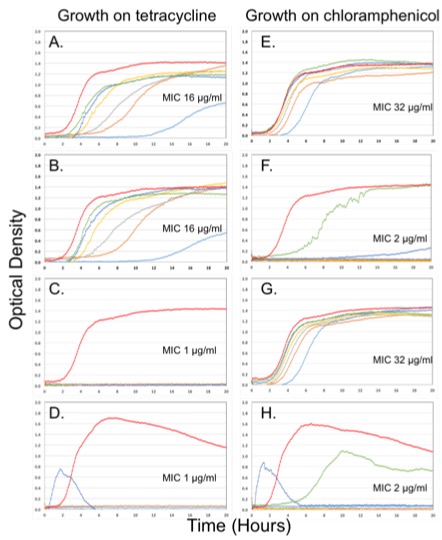

Supplement: Figure S3 — Minimum inhibitory concentrations (MICs) of strains of B. toyonensis on tetracycline (A–D) and chloramphenicol (E–H). (A/E), BCT-7112T; (B/F), BCT-7112Δtet; (C/G), BCT-7112Δcat; (D/H) Rock1-3. The antibiotic concentrations used were (μg/ml): 0, red; 1, green; 2, dark blue; 4, yellow; 8, gray; 16, orange and 32, light blue. The MIC values are shown for each set of growth curves. The experiments were carried out a minimum of three times and representative data show on a linear rather than logarithmic graph to make it easier to compare growth profiles. [file Image3.JPEG]

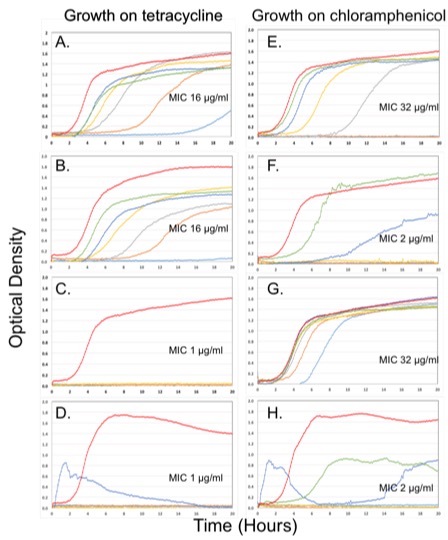

Supplement: Figure S4 — The impact of the gerIC–nucB intergenic region of plasmid pBCT77 on the tetracycline (A–D) and chloramphenicol (E–H) resistance profiles of the following strains: (A/E), BCT-7112(pIGR1); (B/F), BCT-7112Δtet(pIGR1); (C/G), BCT-7112Δcat(pIGR1); (D/H), Rock1-3(pIGR1). The antibiotic concentrations used were (μg/ml): 0, red; 1, green; 2, dark blue; 4, yellow; 8, gray; 16, orange and 32, light blue. The MIC values are shown for each set of growth curves. The experiments were carried out a minimum of three times and representative data show on a linear rather than logarithmic graph to make it easier to compare growth profiles. [file Image4.JPEG]
